# Wavelets-Based Texture Analysis of Post Neoadjuvant Chemoradiotherapy Magnetic Resonance Imaging as a Tool for Recognition of Pathological Complete Response in Rectal Cancer, a Retrospective Study

**DOI:** 10.3390/jcm13237383

**Published:** 2024-12-04

**Authors:** Julia Begal, Edmond Sabo, Natalia Goldberg, Arie Bitterman, Wissam Khoury

**Affiliations:** 1 Department of General Surgery, Carmel Medical Center, Haifa 3436212, Israel; juliyabe@clalit.org.il (J.B.); bitterman@clalit.org.il (A.B.); 2 Department of Human Pathology, Carmel Medical Center, Haifa 3436212, Israel; edmondsa@clalit.org.il; 3 Rappaport Faculty of Medicine, Technion-Israel Institute of Technology, Haifa 3200003, Israel; wisamk@clalit.org.il; 4 Department of Radiology, Carmel Medical Center, Haifa 3436212, Israel

**Keywords:** wavelets, rectal cancer, chemoradiotherapy, pathological complete response

## Abstract

**Background**: Patients with locally advanced rectal cancer (LARC) treated by neoadjuvant chemoradiotherapy (nCRT) may experience pathological complete response (pCR). Tools that can identify pCR are required to define candidates suitable for the watch and wait (WW) strategy. Automated image analysis is used for predicting clinical aspects of diseases. Texture analysis of magnetic resonance imaging (MRI) wavelets algorithms provides a novel way to identify pCR. We aimed to evaluate wavelets-based image analysis of MRI for predicting pCR. **Methods**: MRI images of rectal cancer from 22 patients who underwent nCRT were captured at best representative views of the tumor. The MRI images were digitized and their texture was analyzed using different mother wavelets. Each mother wavelet was used to scan the image repeatedly at different frequencies. Based on these analyses, coefficients of similarity were calculated providing a variety of textural variables that were subsequently correlated with histopathology in each case. This allowed for proper identification of the best mother wavelets able to predict pCR. The predictive formula of complete response was computed using the independent statistical variables that were singled out by the multivariate regression model. **Results**: The statistical model used four wavelet variables to predict pCR with an accuracy of 100%, sensitivity of 100%, specificity of 100%, and PPV and NPV of 100%. **Conclusions**: Wavelet-transformed texture analysis of radiomic MRI can predict pCR in patients with LARC. It may provide a potential accurate surrogate method for the prediction of clinical outcomes of nCRT, resulting in an effective selection of patients amenable to WW.

## 1. Introduction

Half of new diagnoses of rectal cancer are locally advanced (LARC). They are often difficult to treat [1,2]. LARC is defined as cT3-cT4 or N+ (stage II or III) tumors [3,4,5]. Neoadjuvant chemoradiotherapy (nCRT) followed by total mesorectal excision (TME) is the treatment of choice [6,7]. nCRT may induce tumor downstaging, improve sphincter preservation rates, decrease local recurrence, and improve survival rates [8,9]. Moreover, 30–40% of the patients may experience clinical complete response (cCR), while 20% may have a pathological complete response (pCR) [10]. Those may be considered for nonoperative treatment, i.e., watch and wait strategy (WW).

Numerous studies have proposed biomarkers that could predict response to nCRT, such as tumor stage, tumor regression grade, tumor markers, circulating tumor-derived DNA, DNA methylation level, and cancer-related inflammatory markers; however, their accuracy is limited [11,12,13,14].

Current predictors of pCR are poorly defined. A systematic review published in 2016 [15] evaluating predictors of pCR (including clinicopathological variables, imaging modalities, gene expression, mutational, and protein expression analyses) concluded that there were no pCR robust markers. Others reported significant clinical variables that may be associated with pCR; lower tumor grade, lower clinical T and N stage, radiation dose, and delaying surgery post-nCRT by more than 6–8 weeks. Associations, however, were mostly causal [16]. Yet, there is no definite predictor of pCR.

As a new non-invasive imaging technology, radiomics transforms medical imaging into high-dimensional data that can be mined to reveal a large number of quantitative features (including texture, grayscale, wavelet, and fractal), and can combine quantitative features with clinical features, protein genome information, and other information [17,18]. With its advantages, such as being easy to operate, low cost, and high efficiency in capturing the heterogeneity of tumors, it can be used as a new tool for tumor diagnosis and staging, to assess prognosis, and potentially as an imaging modality that can predict tumor response to nCRT [19,20,21,22,23].

## 2. Wavelet Analysis

**Figure 1:**
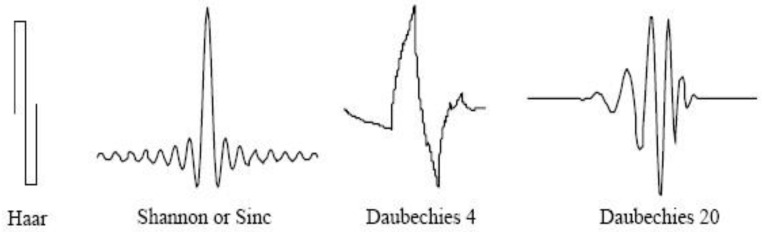
Examples of mother wavelets.

A complete mathematical theory of wavelet analysis has been previously described ([24], Appendix A). Wavelets are a mathematical tool that can be used to extract information from different kinds of data, including—but certainly not limited to—audio signals and images. Sets of wavelets are generally needed to analyze data comprehensively. In brief, a short wave (mother wavelet) is used to scan the image, modify it, and calculate coefficients of similarity between itself and the scanned image at all locations. This mechanism gradually decomposes an image into simpler components. The coefficients of similarity collected from each level of composition were summarized using averages, medians, standard deviations, and maximal values. These collected statistical parameters were used as variables in the statistical analyses for predicting the diagnostic groups (i.e., pCR vs. non-pCR). There are multiple types of mother wavelets that can be used to decompose and analyze the microscopic images and find the best mother wavelets that are able to better predict differences, i.e., pathological response in our study. Some examples of mother wavelets [24] are illustrated in Figure 1.

## 3. Objectives

To identify pCR in rectal cancer patients undergoing nCRT using image analysis. A new method of image analysis based on wavelets-based texture analysis tested against the final pathology diagnosis. This method will hopefully result in nonsurgical treatment, when possible, with preservation of the rectum and offering a better quality of life to the patients.

## 4. Methods

After obtaining an IRB approval following the guidelines of the Helsinki declaration, patients who were treated for low-lying LARC at the department of surgery in Carmel Medical Center, between the years 2018 and 2023 were identified from the departmental database. Patients between 18 and 90 yrs who underwent nCRT for LARC followed by TME were enrolled in the study. In order to protect the identity of the patients, no personal information was used. Data and images were identifiable by a randomly assigned code.

Low-lying rectal cancer was defined as a tumor located between 1 and 10 cm from the anal verge, measured by rigid proctoscopy.

Routinely, at our department, rectal tumors are evaluated by digital rectal exam (DRE), rigid proctoscopy, colonoscopy, and abdominal and chest computerized tomography. Patients also undergo pelvic MRI to determine the local tumor stage. Those with T3-4NxM0 or Tx/N1M0 radiologic tumor stage are amenable to nCRT. Upon completion of nCRT, patients are evaluated again to assess response to radiation, tumor regression, or progression. Reassessment includes repeated DRE, proctoscopy, and MRI, 6–8 weeks after nCRT. The patients who experience cCR are considered for the WW strategy. Otherwise, surgery was scheduled 10–11 weeks after nCRT. The patients who did not undergo repeated MRI after nCRT or pursue a WW strategy were excluded from the study. Those who underwent surgery and their MRI images pre- and post-nCRT were available consisted the study group.

The MRI images were extracted from the radiology archives using the PACS digital system (Phillips). The collected MRI images were electronically filed and reviewed on a high-resolution plasma screen. The tumor site was determined based on pre- and post-nCRT MRI. The best representative views of the tumor in post-nCRT images were selected by an experienced MRI body radiologist. For that purpose, we used T2, DWI (diffusion-weighted imaging), and ADC (apparent diffusion coefficient) modalities sequences. Regions of interest (ROI) on the section containing the largest proportion of tumor that was most representative were manually selected. Coregistration tool in the Carestream pacs was used to apply different sequences. T2-weighted images converted to bitmap gray levels, saved as bmp formats and were used for the analysis. The Matlab R2015a (Mathworks, USA) program was used to perform the wavelet analysis of the images.

Wavelet transformation operates by computing inner products between a signal (f(x)) and analysis functions derived by rescaling and translation from a wavelet function, often referred to as the “mother wavelet”. The formulation below is that given by Unser (1996) where Ψ_a,b_ is the “mother wavelet” and a and b are rescaling and translation parameters, respectively (see Appendix A).

$$\Psi_{a,b}(t)=1/\sqrt{a}\Psi (t-b/a)$$

### 4.1. Variables

**Figure 2:**
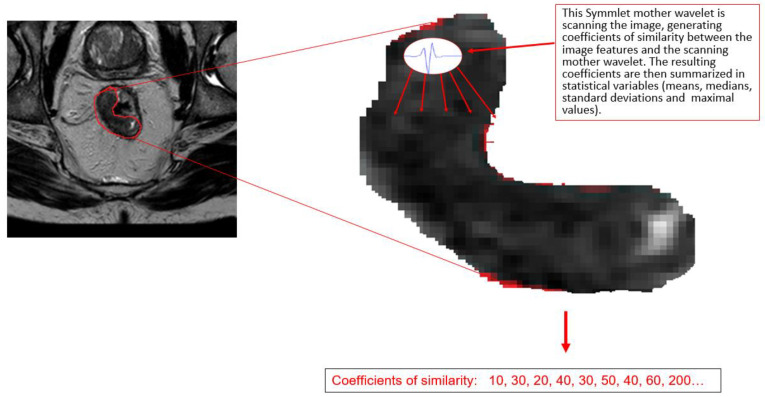
Illustration of the workflow of regions of interest (ROI) scanning from a magnetic resonance image by a mother wavelet.

The average, median, maximal, and standard deviations of the coefficients of similarity (Figure 2) generated by multiple mother wavelets were computed and used to compute a discriminant function able to differentiate between complete and noncomplete response, based on histopathology outcomes for each case. Independent variables and their coefficients were used to build a predictive discriminant score (DS) for pCR. We used 10 different mothers wavelets (db1, db2, db4, coif1, coif5, bior2.2, bior3.3, sim2, sim3, and sim5). Each wavelet we used has 12 variables (see Appendix A).

### 4.2. Statistical Analysis

After testing the variables for normality using the Kolmogorov—Smirnov test, a univariate analysis was performed using the Student’s *t*-test, followed by a multivariate discriminant regression analysis in order to single out independent wavelets’ predictors of pCR. Two-tailed *p*-values of 0.05 or less were considered to be statistically significant. The independent variables that were detected by the discriminant regression model and their coefficients of regression were used to build a predictive DS for pCR. The best DS cutoff score was found using a receiver operating characteristic (ROC) analysis. The leave-one-out cross-validation was also used. The statistical analysis was performed using the IBM-SPSS version 22 [25].

## 5. Results

### Clinical and Pathological Data

Twenty-two patients met the inclusion criteria. Their mean age was 58.1 ± 12.7 yrs. Seven patients experienced a pCR. The patients and tumor characteristics are presented in Table 1.

More female patients were reported in the pCR group. There were no significant differences between groups. Also, preoperative treatment protocols were comparable. Most patients in the pCR group received TNT. Of note, the number of harvested lymph nodes distributed equally between groups, probably represents a consistent surgical procedure in all patients.

A large number of variables in the studied images, using the wavelets analysis, were compared between pCR and non-pCR groups. Near or significantly different variables in a univariate analysis are listed in Table 2. In the multivariate regression model, significantly different variables between groups are presented in Table 3. Those variables may be predictors of pCR.

Based on the above independent wavelets’ predictors and their regression coefficients, the following formula of the DS for each patient was computed:

DS = −541.1768021 + (0.2456044 × db2dec2sd) + (1.1709781 × bior33dec1max) + (0.0399379 × bior33dec3mn) − (0.0691719 × sym5dec2mn)

**Figure 3:**
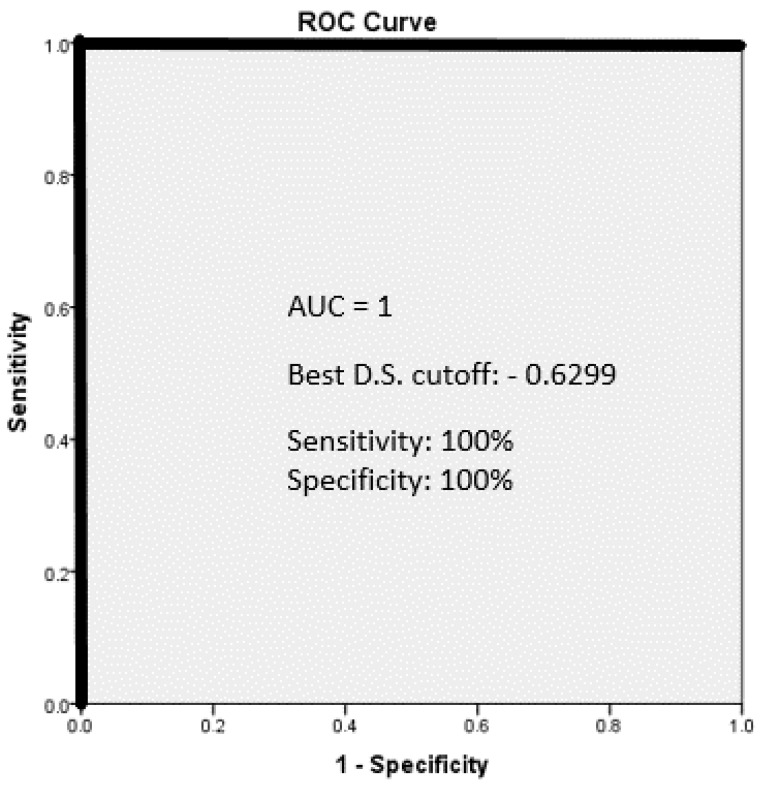
A receiver operating characteristic (ROC) analysis of the discriminant score analysis.

The DS was used to predict pCR in these patients. In order to detect the best DS cutoff for the best pCR predictive values, an ROC analysis of the DS was performed as shown in Figure 3.

**Figure 4:**
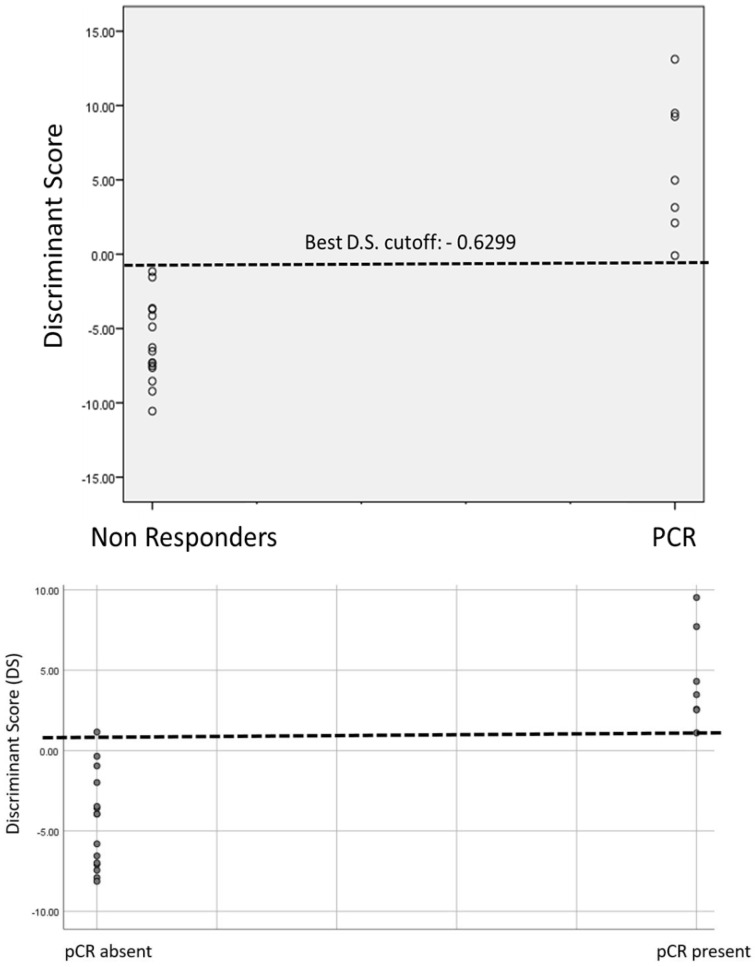
Discriminant score values in relation to pathological complete response (pCR) and the best predictive cutoff.

DS values in relation to pCR and the best predictive cutoff are shown in Figure 4.

The best DS cutoff is −0.6299. This cutoff was able to correctly predict pCR (sensitivity) in 100% of the cases and to predict nonresponsiveness (specificity) in 100% with a total accuracy of 100%. The positive predictive value (PPV) of the cutoff is 100% and the negative predictive value is 100%.

The leave-one-out cross-validation results provided by the model showed a sensitivity of 100%, specificity of 100%, and total accuracy of 100%.

## 6. Discussion

The gold standard treatment of LARC is nCRT followed by TME [25,26]. Surgical resection, however, is associated with significant perioperative morbidity and mortality. Permanent colostomy also is constructed in some patients.

nCRT may improve locoregional control and downstage rectal tumor [27]. A total of 15% to 27% of the patients experience a pCR [28,29]. Usually, patients undergoing nCRT are reevaluated for clinical response. If cCR is achieved, i.e., absence of disease per digital rectal exam, endoscopy, and MRI, the patients are amenable for the WW approach, which offers a novel management strategy, allowing organ preservation. However, up to 25% of the patients with cCR may develop disease regrowth [10]. Therefore, accurate diagnosis of pCR rather than cCR is needed to allow patients as well as doctors to take advantage of the WW strategy safely.

MRI is commonly used to triage patients to surveillance programs after nCRT. However, its role in the prediction of treatment response to nCRT is unclear [5,6]. The main challenges to the widespread clinical use of MRI are the intrinsic limitation of imaging itself for identifying treatment-induced changes and observer dependency. Both, missing pCR and overdiagnosing pCR, are reported. Diffusion weight imaging (DWI)-MRI, a functional MRI component, may improve MRI performance and can be used to investigate meaningful biological properties such as tissue cellularity and water content induced after nCRT [30,31].

A new method, radiomics, using MRI images as a predictor of rectal cancer response to nCRT has also been reported [32]. In particular, wavelet analysis is a new non-invasive technology that may play a role in evaluating post-treatment MRI by capturing the heterogeneity of tumors, thus forming a successful tool for predicting the response to nCRT [20,21,22,23,24]. Radiomics techniques can detect patterns in the data beyond those appreciated by the radiologic eye and ultimately improve response classification. Shin et al. built a radiomics model from post-treatment MRI features and yielded an area under the curve (AUC) of 0.82 [33]. However, the post-treatment analysis did not provide direct guidance on early intervention. We used a similar technique, the wavelet analysis, to define pCR. It successfully detected pCR by combining four wavelet variables in a regression-based model. It predicted the pCR with an accuracy rate of 100%, a sensitivity of 100%, a specificity of 100%, and a PPV and NPV of 100%. The leave-one-out validation method also revealed significantly high predictive values.

Wavelet analysis can evaluate tumor response through several pathways. It may estimate changes in the texture or spatial distribution of tumor tissues. By decomposing the MRI images into different scales or levels, wavelet analysis can highlight subtle changes in the tumor’s appearance, such as changes in shape, size, or intensity of distribution. Therefore, defining a representative MRI image by an experienced radiologist was crucial.

Wavelets may also analyze the perfusion characteristics of the tumor. It can be used to extract the temporal dynamics of contrast enhancement in MRI images, which can provide insights into the tumor’s blood flow and vascularization. Changes in the wavelet coefficients associated with perfusion parameters, such as blood volume or flow rate, can indicate treatment response or the presence of residual tumor tissues. Chemotherapy and radiotherapy enhance water diffusivity in the tissue, possibly indicative of tumor cell death or a reduction in cell density, making the tumor environment more permeable. Those tissue changes may translate into different radiologic features of MRI [30] and eventually into different coefficient variables. Our study showed that four coefficient variables, representing a tissue change were consistently associated with pCR. Similarly, DWI-MRI which measures the magnitude of diffusion provides valuable information about tissue properties in biological systems and has been proven to be a quantitative biomarker that may guide clinicians on tissue changes post-nCRT [30,31]. Those changes may again translate into differences in wavelet measurements.

Finally, wavelet analysis can extract features or biomarkers from the MRI images that can be used for quantitative analysis and prediction of treatment outcomes [34]. By analyzing the wavelet coefficients at different scales or resolutions, statistical or machine-learning algorithms can be applied to identify patterns or correlations between the extracted features and clinical parameters, such as tumor size, stage, or response to treatment. This allows developments of predictive models for treatment response or prognosis. Herein, we showed that MRI images in patients with pCR have common overlapping coefficients that may represent a true pathological regression of the tumor with an accuracy of 95%. A large-scale image may allow the creation of a comprehensive model of wavelet analysis based on a large number of variables that can potentially predict response to treatment based on post-nCRT MRI images.

To our knowledge, this is the first study that used wavelet analysis of post-nCRT MRI to predict rectal cancer response to nCRT. Since post-nCRT MRI is expected to differentiate between complete, partial, or no response, we chose to study differences between MRI images given the final pathology results. In fact, in this pilot study, we are trying to identify variables that are pathognomonic for pCR. Truly, we were able to find characteristic variables associated with pCR.

Despite representing promising results, the study has several limitations. First, the retrospective nature of the study. Second, small sample size. Therefore, the results presented above may be considered as a proof of concept that needs to be further validated in larger study groups. Third, the ROIs were delineated manually. A single experienced body radiologist manually outlined all the tumors. Despite a certain level of consistency, this method presents certain drawbacks as it is not possible to achieve complete agreement with other potential readers. As a result, the reliability of the variables derived from these manually drawn ROIs may be compromised. Fourth, only tumor mass was evaluated, mesorectum and lymph nodes were not investigated. Fifth, the study raises important issues that must be considered in all radiomics research. It proposes a predictive marker for a short-term clinical event, post-nCRT pCR detected with post-nCRT MRI. This seems a sensible and clinically meaningful approach and aligns with current staging and treatment pathways. An important limitation, however, is that non-imaging predictive markers, such as digital rectal exams and endoscopies, are not included. As noted above, the assessment of pCR is based not just on MRI but also on clinical and endoscopic assessment. Future work must incorporate all relevant clinical predictors. Thus, there is a long way to go before such models can be considered for clinical use.

## 7. Conclusions

Our study demonstrated that wavelet-based texture analysis of MRI images after nCRT can successfully predict pCR in patients with LARC, with an impressive accuracy of 100%. Therefore, the method presented in this study may provide a potential surrogate for the accurate prediction of the clinical outcomes of nCRT, resulting in a more effective selection of patients for the WW strategy, allowing preservation of the rectum, thus conferring better quality of life in this group of patients.

## Figures and Tables

**Table 1:** Patients and tumor characteristics.

		pCR Present Group *n* = 7 (31.8%)	pCR Absent Group *n* = 15 (68.2%)	*p* Value
Age (Mean ± SD)		56 ± 13.1	59 ± 12.9	0.6
Gender (%)	Female	5 (71.4%)	4 (26.7%)	0.06
	Male	2 (28.6%)	11 (73.3%)	
BMI (mean ± SD)		30.4 ± 9	26.7 ± 3.6	0.17
cTNM (%)	T3N0	0 (0%)	2 (13.3%)	
	T3N1	4 (57.1%)	7 (46.6%)	
	T3N2	2 (28.6%)	4 (26.7%)	
	T4N0	0 (0%)	1 (6.7%)	
	T4N1	1 (14.3%)	0 (0%)	
	T4N2	0 (0%)	1 (6.7%)	
Anal verge distance (mean ± SD)		6 ± 3	7 ± 2	0.64
Radiation%	Short course	0 (0%)	4 (26.7%)	
	Long course	7 (100%)	11 (73.3%)	
Total Neoadjuvant Treatment%		6 (85.7%)	8 (53.3%)	0.16
Interval between radiation therapy and surgery (weeks) (mean ± SD)		14.1 ± 9.5	11.6 ± 6.2	0.53
Surgical approach%	Laparoscopic	4 (57.1%)	11 (73.3%)	
	Robot-assisted	3 (42.9%)	4 (26.7%)	
Number of harvested lymph nodes (mean ± SD)		14 ± 3	14 ± 7	0.77
Involved lymph nodes%		0 (0%)	5 (33.3%)	
Pathology Stage (%)	T0N0	7 (100%)	0(0%)	
	T1N0	0 (0%)	2 (13.3%)	
	T1N1	0 (0%)	1(6.7%)	
	T2N0	0 (0%)	5 (33.3%)	
	T2N1	0 (0%)	1 (6.7%)	
	T3N0	0 (0%)	1 (6.7%)	
	T3N1	0 (0%)	5 (33.3%)	

pCR—pathological complete response, SD—standard deviation, BMI—body mass index.

**Table 2:** Univariate wavelet analysis of variables that are near or significantly different between groups.

Wavelets Variable	pCR Present (Mean ± SD)	pCR Absent (Mean ± SD)	*p* Value
db1dec1max	429 ± 1.0	427.93 ± 2.043	0.12
db1dec1sd	87.36 ± 17.85	72.46 ± 17.62	0.08
db1dec2max	866.46 ± 1.86	860.27 ± 5.51	0.001
db2dec1sd	88.53 ± 19.2	75.11 ± 18.64	0.13
db2dec2sd	191.107 ± 26.92	165.39 ± 28.33	0.05
coif1dec1sd	88.295 ± 20.07	71.68 ± 20.3	0.08
coif5dec2max	869.22 ± 0.82	867.83 ± 2.7	0.08
bior33dec1max	428.362 ± 1.88	424.91 ± 7.2	0.1
bior33dec3mn	846.84 ± 226.27	819.92 ± 215.82	0.79
sym5dec2mn	508.66 ± 107.91	516.83 ± 106.97	0.87

pCR—pathological complete response.

**Table 3:** Multivariate analysis results (discriminant regression model): independent predictors of pathological complete response.

Independent Wavelets Variable	Beta (Slopes)	*p* Value
db2dec2sd	0.2456044	<0.001
bior33dec1max	1.1709781	<0.001
bior33dec3mn	0.0399379	<0.001
sym5dec2mn	0.0691719	<0.001
Constant	−541.1768021	<0.001

## Data Availability

The original contributions presented in the study are included in the article, further inquiries can be directed to the corresponding author.

## Appendix A

**Wavelets mathematics in a nutshell:**

In wavelets analysis, two functions—the scaling and the wavelet functions—are fundamental components which are needed for performing the wavelets multiresolution scanning and analysis of an image which is considered to be a two-dimensional digital (discrete) signal. The parameters and variables of these functions are integrated along the progressively scanned image signal. These wavelet functions are sometimes named mother wavelet which forms the core function used in wavelet analysis, the father wavelet which captures the low-frequency components, and the term baby wavelet that is less commonly used and may refer to specific instances of wavelet functions derived from the mother wavelet. Here is a more detailed explanation of the wavelet functions:

The first function is called the scaling function (also called father wavelet) as follows:

$$\emptyset \left(t\right)=\sum_{k}h_{k}\emptyset (2t-k)$$

This scaling function, ø(t), is considered to be a low-pass filter (allowing the low frequencies of a signal to pass) and is therefore representing the coarse approximation of the smooth (low frequency) components of the signal, also called the coarse approximation of the signal, emphasizing the main dominant pattern which represents the 2D image signal. The coefficients *h_k_* can be considered the taps of a low-pass filter. The t parameter is the special point where the scanning wavelet is progressing along the signal and the 2t − k expression refers to the degree of shifting of the scanning wavelet along the signal.

When the signal is convolved with this scaling function/filter followed by a down-sampling approach, the result is a down-sampled version of the signal which retains the low frequency components. This operation effectively performs a low-pass filtering of the signal.

The second component of the wavelet analysis is the wavelet function (also called mother wavelet) as follows:

$$\psi \left(t\right)=\sum_{k}g_{k}\phi \left(2t-k\right)$$

The wavelet function, ψ(t), is considered to be a high-pass filter, capturing the detail or high frequency component of the signal, highlighting the sudden changes or discontinuities, and for practical purposes, representing the noisy component of the signal.

The coefficients *g_k_* can be considered the taps of a high-pass filter. Convolution of the signal with this filter followed by down-sampling yields the high-frequency components of the signal, effectively capturing the details within the signal.

The scaling and the wavelet functions are related to each other through the concept of multiresolution analysis. The scaling function generates the approximation spaces while the wavelet function generates the detail spaces. Specifically, the wavelet function can be derived from the scaling function and both functions are associated with a specific set of filter coefficients. Moreover, any signal can be reconstructed from its wavelet coefficients (approximation and detail) using both the scaling and the wavelet functions.

In wavelet theory, the terms “mother wavelet”, “father wavelet”, and “baby wavelet” refer to different concepts related to wavelet functions and their roles in signal decomposition and reconstruction.

In many wavelet systems, the scaling function and the wavelet functions are **orthogonal** to each other. This means that the inner product of the two functions over their domain is zero.

**Orthogonal wavelets** have the property that their inner product is zero for different wavelets. This means they are mathematically independent of each other.

The advantages of the orthogonal wavelet functions are as follows:

Simplicity in computation: Fast algorithms (like the Fast Wavelet Transform) can be easily implemented.

Energy conservation: Orthogonal wavelets preserve the energy of the signal, which is useful in reconstruction.

Good for certain types of signals: They perform well on smooth signals and are effective for compression.

Examples of orthogonal wavelets include Haar, Daubechies, and Coiflets.

Haar Wavelet: The simplest wavelet, characterized by a step function. It is often used for quick, straightforward applications.

Daubechies Wavelets: These wavelets are defined by their number of vanishing moments (see comment below). They are widely used in various applications due to their compact support and good performance.

Coiflets: A family of wavelets with higher vanishing moments than Daubechies wavelets, providing smoother results.

**Comment:** Vanishing moments refer to the properties of wavelets that determine their ability to represent certain types of functions or variations in a signal.

Specifically, the nth vanishing moment of a wavelet indicates that the wavelet can capture variations of order up to *n* − 1 in a function.

Definition: A wavelet has *n* vanishing moments if the integral of the wavelet function multiplied by x^k^ is zero for all k = 0, 1, …, *n* − 1

Mathematically, this means

$$\int_{-\infty }^{\infty }\psi (x)x^{k}dx=0\;\;\;\;\;for\;k=0,1,\ldots ,\;n-1$$

where ψ(t) is the wavelet function.

Implication: The more vanishing moments a wavelet has, the better it can represent polynomial behavior up to a certain degree. For example, a wavelet with two vanishing moments can effectively represent linear variations, while one with three vanishing moments can handle quadratic variations.

Applications: Vanishing moments are important in applications like signal processing, compression, and denoising, as they determine how well a wavelet can capture features in the data without introducing artifacts.

In summary, vanishing moments reflect a wavelet’s ability to capture specific types of information in a signal, influencing its effectiveness for different tasks.

**Biorthogonal wavelets** consist of two different sets of wavelets (unlike the orthogonal wavelets that have one set of father wavelets and one set of mother wavelets). The two sets of biorthogonal wavelet forms one set for analysis and a second set for synthesis. They do not have to be orthogonal to each other. In the biorthogonal wavelets, analysis and synthesis refer to the dual role these wavelets play in the wavelet transform process.

Analysis and Synthesis Explained

Analysis Wavelet: This wavelet is used to decompose a signal into its wavelet coefficients. Think of this as taking a photograph of a landscape. The analysis wavelet captures different features of the signal at various scales, allowing us to see details and patterns.

Synthesis Wavelet: After analysis, the synthesis wavelet is used to reconstruct the original signal from the wavelet coefficients. This is akin to assembling a puzzle. The synthesis wavelet combines the coefficients back into a cohesive whole, ensuring that the original signal can be accurately reconstructed.

This separation allows for greater flexibility in design. For instance, the analysis wavelet can be optimized to best capture the signal’s features, while the synthesis wavelet can be tailored to improve the reconstruction quality.

**The advantages of the biorthogonal wavelet functions are as follows:**

**Flexibility:** The analysis and synthesis wavelets can be tailored for specific applications.

**Symmetry:** Many biorthogonal wavelets can be symmetric, which can improve the appearance of reconstructed signals.

**No loss of information:** Biorthogonal wavelets can provide perfect reconstruction while allowing for more degrees of freedom in the design.

In essence, the concept of having both analysis and synthesis wavelets in biorthogonal systems enhances the ability to effectively decompose and reconstruct signals, making them a powerful tool in various signal processing applications.

**Examples of biorthogonal (Bior) wavelets include the following:**

Biorthogonal splines such as bior2.2 and bior3.3. These wavelets, often referred to as B-spline wavelets, provide both an analysis and a synthesis wavelet. They are symmetric and have flexible design options. These are used for applications requiring perfect reconstruction, such as image compression. In essence, the concept of having both analysis and synthesis wavelets in biorthogonal systems enhances the ability to effectively decompose and reconstruct signals, making them a powerful tool in various signal processing applications.

The terms “bior2.2” and “bior3.3” refer to specific biorthogonal wavelet families used in signal and image processing. The key differences between them are as follows:

**Bior2.2:**

Structure: It has two vanishing moments for both the scaling function and the wavelet function, which means it can capture quadratic variations in data.

Symmetry: This wavelet is symmetric, which is beneficial for applications where preserving phase information is important.

Applications: Often used in image compression and denoising tasks.

**Bior3.3:**

**Structure**: It has three vanishing moments for the wavelet function and three for the scaling function. This makes it better suited for capturing cubic variations in data.

Symmetry: Like bior2.2, it is also symmetric, providing similar advantages in terms of phase preservation.

Applications: It tends to provide better performance in terms of compression and detail preservation, making it preferable for certain types of images or signals.

Summary:

In essence, the choice between bior2.2 and bior3.3 depends on the specific characteristics of the data one is working with and the desired level of detail and performance in the analysis or processing tasks.

**Non-orthogonal wavelets** do not have the orthogonality property. They can be derived from various approaches and are often less commonly used in practice.

The advantages of the non-orthogonal wavelet functions are as follows:

Flexibility in design: They allow for a wider range of wavelet shapes and can be optimized for specific problems.

Potentially better for specific applications: In some contexts, non-orthogonal wavelets might capture certain features of a signal more effectively.

**Examples of non-orthogonal wavelets include** Symlets and Mexican Hat Wavelets (Ricker Wavelet).

**Symlets:** A modification of Daubechies wavelets that may not be strictly orthogonal but are designed to be symmetric, making them useful in certain contexts where symmetry is beneficial.

**Mexican Hat Wavelet (Ricker Wavelet):** A second derivative of a Gaussian function, commonly used in signal processing, especially in feature extraction.

**The scaling and wavelet function slightly differ among the different types of wavelets.**

For example:

**The Daubechies and related orthogonal Wavelet system:**

Scaling function: The Daubechies scaling function is given by

$$\emptyset \left(t\right)=\sum_{k}h_{k}\emptyset (2t-k)$$

Wavelet function: The Daubechies wavelet function is given by

$$\psi \left(t\right)=\sum_{k=0}^{N-1}g_{k}\phi (2t-k)$$

where the *g_k_* coefficients are the wavelet filter coefficients, related to the scaling coefficients by the following relation:

$$g_{k\;}={\left(-1\right)}^{k}h_{N-1-k}$$

**The Symlet Wavelet system:**

Scaling function: Symlet wavelets are a modified version of Daubechies wavelets designed to be more symmetric. Their scaling functions maintain a smoother shape compared to the Haar wavelet.

**The Coiflet wavelet system:**

Scaling function: Coiflets have scaling functions that are smoother and possess more vanishing moments. They are designed to have both a certain degree of symmetry and a specific number of vanishing moments.

Wavelet function: The Coiflets wavelet function inherits the properties of the scaling function, allowing for effective representation of signals.

**The Biorthogonal (Bior) Wavelet system:**

The scaling function: Bior wavelets have two sets of scaling functions (one for the analysis and one for the synthesis). They can be designed to be symmetric or asymmetric, depending on the chosen parameters.

The Wavelet function: Like the scaling function, Bior wavelets also have associated wavelet functions that can be constructed to achieve specific properties as symmetry.

**Morlet wavelet system:**

Scaling function: The Morlet wavelet does not have a traditional scaling function like others. It is defined using a complex sinusoid modulated by a Gaussian envelope.

Wavelet function: The Morlet wavelet function is given by

$$\;\psi (t)=e^{2\pi if₀t}e^{-t^{2}/2\sigma^{2}}$$

where f_0_ is the central frequency and σ controls the width of the Gaussian.

**Mexican Hat wavelet system:**

Scaling function: The Mexican Hat (Ricker wavelet) wavelet does not have a scaling function in the classical wavelet sense. It is derived from the second derivative of a Gaussian function.

Wavelet function: the Mexican Hat wavelet is expressed as

$$\;\psi (t)=e^{2\pi if₀t}e^{-t^{2}/2\sigma^{2}}$$

The function resembles a sombrero shape and is useful for edge detection in images.

Each of these wavelet types has unique properties that make them suitable for different applications in signal processing and other fields.

**Decomposition Levels of the signal by the wavelet:**

In the context of wavelet transforms, “level” refers to the number of times the signal has been decomposed into its wavelet components. Each level of decomposition captures different frequency components of the signal, providing a hierarchical representation of its information.

**Level 1**: The signal is decomposed once. This typically results in two sets of coefficients: the approximation coefficients (low-frequency components) and the detail coefficients (high-frequency components).

**Level 2**: The approximation coefficients from Level 1 are further decomposed. This process yields a new set of approximation and detail coefficients, providing a finer granularity of the signal’s features.

**Level 3**: The decomposition continues, with the approximation coefficients from Level 2 being decomposed again, leading to even more detailed coefficients.

**Hierarchical Representation**: Each level captures progressively finer details of the signal. Higher levels emphasize higher frequency components, while lower levels capture broader trends or features.

**Applications**: Different levels are useful for various applications. For instance, in signal compression, lower levels can capture the most important information while higher levels can be used for more detailed analysis or noise reduction.

In summary, the wavelet level indicates how many times the signal has been broken down into its wavelet components, with higher levels providing more detailed information about the signal’s structure.

In the context of Daubechies wavelets, the scaling function and wavelet function evolve through the decomposition levels, reflecting the different frequency components of the signal.

**Level 1 Decomposition:**

**Scaling Function ϕ(t)**: This function captures the low-frequency (smooth) components of the signal. It is used to compute the approximation coefficients.

**Wavelet Function ψ(t)**: This function captures the high-frequency (detail) components of the signal. It provides the detailed coefficients.

At this level, the signal is split into two components as follows:

**Approximation**: Represents the coarse features.

**Detail**: Captures the finer features.

**Level 2 Decomposition:**

**New Scaling Function**: The scaling function from Level 1 is used to generate a new scaling function, often denoted as ϕ2(t). It will be narrower and oscillate more rapidly, capturing more details of the signal.

**New Wavelet Function**: Similarly, the wavelet function from Level 1 produces a new wavelet function, ψ2(t). This wavelet function will also be more oscillatory than the Level 1 wavelet, capturing finer details.

At this level, the approximation coefficients from Level 1 are decomposed again, leading to the following:

**New Approximation**: A more refined representation of the signal’s low-frequency components.

**New Detail**: A more intricate representation of the signal’s high-frequency components.

**Level 3 Decomposition:**

**Further New Scaling Function**: The process continues, leading to a third scaling function, ϕ3(t), which is even narrower and captures the very smooth features of the signal at this level.

**Further New Wavelet Function**: The wavelet function ψ3(t) becomes even more oscillatory, allowing for a detailed representation of rapid changes or noise in the signal.

At this level, the approximation from Level 2 is decomposed again, resulting in the following:

**New Approximation**: A further refined low-frequency representation.

**New Detail**: Captures even higher-frequency components than Level 2.

**Summary of Changes:**

**Level 1**: Broadest, captures major trends.

**Level 2**: More detailed, captures nuances from Level 1’s approximation.

**Level 3**: Most detailed, capturing rapid changes and fine structures in the signal.

This hierarchical decomposition is one of the key strengths of wavelet analysis, allowing for both global and local feature representation. Each level provides a different perspective on the signal’s structure, with the scaling and wavelet functions evolving to reflect these changes.

**Explanation of the abbreviations of the variables that were extracted from the wavelet analysis in this study:**

The first part of the name represents an abbreviation of the wavelet type. The second part represents the level of decomposition. The third and last part of the variable name represents the statistics calculated from the approximation coefficients of similarity of the wavelets.

For example, the following variable abbreviation **dbdec1med** means the following:

**db:** means the wavelet type.

**dec1**: means level 1 (first) decomposition of the signal.

**med:** the median of the approximation coefficients of similarity that resulted from the scaling and wavelet functions.
